# Analysis of the clinical efficacy of laparoscopic middle pancreatectomy in the treatment of benign or low-grade malignant tumors of the pancreas

**DOI:** 10.3389/fonc.2023.1231647

**Published:** 2023-11-02

**Authors:** Yan Liao, Wei Zhou, Manxiong Dai, Jie Zhou, Yi Wang, Xiangyu He, Yi Liu, Wei Cheng

**Affiliations:** ^1^ Department of Hepatobiliary Surgery, Hunan Provincial People’s Hospital, The First Affiliated Hospital of Hunan Normal University, Changsha, Hunan, China; ^2^ Translational Medicine Laboratory of Pancreas Disease of Hunan Normal University, Changsha, China; ^3^ Department of Hepatobiliary Surgery, Yueyang People’s Hospital, Affiliated Hospital of Hunan Normal University, Yueyang, Hunan, China; ^4^ Department of General Surgery, The Third People’s Hospital of Hunan Province, Yueyang, Hunan, China

**Keywords:** laparoscopic, middle pancreatectomy, postoperative pancreatic fistula, endocrine function, exocrine function

## Abstract

**Objective:**

The aim of this study was to investigate the clinical efficacy of laparoscopic middle pancreatectomy in the treatment of benign and junctional tumors of the pancreas.

**Methods:**

Retrospective analysis of basic data, tumor diameter, statistical analysis, and evaluation of efficacy-related indicators such as operative time, intraoperative bleeding, pathological findings, postoperative hospital stay, postoperative pancreatic fistula incidence, and pancreatic endocrine function was carried out on 17 patients diagnosed with benign or low-grade malignant tumors of the pancreas and laparoscopic middle pancreatic resection from January 2018 to January 2023 at the First Affiliated Hospital of Hunan Normal University.

**Results:**

A total of 17 patients were screened. There were eight males and nine females; mean age was 42.8 ± 17.4 years (range: 15–69 years); BMI was 22.6 ± 2.5 kg/m2 (range: 18.4–27.5 kg/m2), and the tumor size was 3.4 ± 1.2 cm (range: 1.5–5.5 cm). Preoperative glycan antigen CA19-9 was negative and CA125 was negative. Surgical time was 393.2 ± 57.9 min; intraoperative bleeding was 211.7 ± 113.9 ml; tumor diameter size was 3.4 ± 1.2 cm; postoperative admission time was 19.4 ± 7.6 days; postoperative pancreatic fistula (POPF) grading was 17 cases, including nine cases of A-grade fistula, three cases of B-grade fistula, and none of C-grade fistula; postoperative pathology results were five cases of plasmacytoma, three cases of mucinous cystadenoma, four cases of SPN (solid pseudopapillary neoplasm), one case of Intraductal Papillary Mucinous Neoplasm (IPMN), three cases of pancreatic Neuroendocrine Neoplasm (pNEN), one case of inflammatory myofibroblastic osteoblastoma. All cases did not develop pancreatic origin diabetes or exacerbation of previous diabetes, and no cases presented symptoms of exocrine insufficiency such as dyspepsia and diarrhea.

**Conclusion:**

Laparoscopic middle pancreatectomy is safe and feasible in the treatment of benign or low-grade malignant tumors in the body of the pancreatic neck and is not accompanied by increased risk of intraoperative and postoperative complications and endocrine dysfunction of the pancreas.

## Introduction

The surgical treatment of pancreatic tumors includes pancreaticoduodenectomy (PD), distal pancreatectomy (DP), and total pancreatectomy. However, it is a hot topic of current research to preserve the organ function and maintain physiological access while reducing the incidence of postoperative complications and quality of life after surgical intervention for benign or malignant tumors of the pancreas. For benign and junctional tumors of the pancreas, restrictive pancreatic resection procedures, namely, middle pancreatectomy (MP), local enucleation, and subtotal resection with preservation of the mid-pancreatic segment, have been proposed to obtain better functional outcomes without compromising the radicality of the tumor in the treatment of benign or low-grade malignant tumors (1, 2). However, in the treatment of benign and junctional tumors of the pancreas, laparoscopic MP offers less experience to draw from because of its surgical difficulty and the limited number of cases. To this end, investigations were hereby carried out over the short- and long-term outcomes of organ-preserving laparoscopic pancreatectomy for benign or low-grade malignant pancreatic tumors performed at our center, and experiences of the center with laparoscopic MP were provided. In view of this, a retrospective analysis was conducted to summarize the short- and long-term outcomes of MP for benign or low-grade malignant pancreatic tumors performed at a single center over the past 5 years.

## General information

### Inclusion criteria

The indications for MP include the following: (1) tumors located in the pancreatic neck, (2) benign junctional or low-grade malignant tumors without vascular invasion, and (3) tumors <5 cm in diameter (3). However, this procedure is not suitable for those with traumatic rupture of the body of the pancreatic neck (4). Commonly, benign or low-grade malignant tumors such as neuroendocrine tumors, plasmocytic cystadenomas, mucinous cystadenomas, non-invasive intraductal mucinous tumors, solid pseudopapillary tumors, and isolated metastatic lesions are included.

### Patient information

All patients who suffered from benign or low-grade malignant pancreatic tumors and underwent laparoscopic MP at our institution from January 2018 to January 2023 were hereby included according to the above criteria. All of them underwent preoperative-enhanced computed tomography (CT) or magnetic resonance imaging scans of the abdomen. The diagnosis was carried out by a multidisciplinary team specialized in pancreatic surgery, and an individualized treatment plan was developed. All patients signed an informed consent form, and the study was approved by the ethical review committee of the First Affiliated Hospital of Hunan Normal University. The characteristics of all patients, surgical features, and intraoperative and postoperative outcomes were retrospectively analyzed. The follow-up was conducted until January 2023.

(**Table 1**: Demographic characteristics, surgery, and pathology).

**Table 1:** Demographic characteristics, surgery, and pathology.

Patient (case)	Age (years)	Sex	BMI (kg/m^2^)	Ca19–9 (+or–)	CA125 (+or–)	Surgery	Diagnosis
1	45	Male	23.8	–	–	LMP&PJ	pNEN
2	55	Female	22.4	–	–	LMP&PJ	SPN
3	22	Female	24.1	–	–	LMP&PJ	SCN
4	23	Female	23.1	–	–	LMP&PJ	SPN
5	55	Male	19.4	–	–	LMP&PJ	pNEN
6	69	Female	21.1	–	–	LMP&PJ	SCN
7	26	Female	22.3	–	–	LMP&PJ	pNEN
8	58	Female	20.8	–	–	LMP&PJ	SCN
9	51	Male	21.7	–	–	LMP&PJ	inflammatory myofibroblastoma
10	68	Male	23.1	–	–	LMP&PJ	SCN
11*	50	Male	27.5	–	–	LMP&PJ	SCN
12	34	Male	23.3	–	–	LMP&PJ	SPN
13	58	Female	21.7	–	–	LMP&PJ	MCN
14	27	Male	24.6	–	–	LMP&PJ	MCN
15	15	Male	19.8	–	–	LMP&PJ	SPN
16	23	Female	18.4	–	–	LMP&PJ	MCN
17	50	Female	28.1	–	–	LMP&PJ	IPMN

* It’s diabetic.

### Surgical methods and procedures

Position of the patient: the patient was lying supine in the split-leg position, the main incision was located on the right side of the patient, the assistant was on the left side of the patient, and the supporting hand was between the legs of the patient.

**Figure 1:**
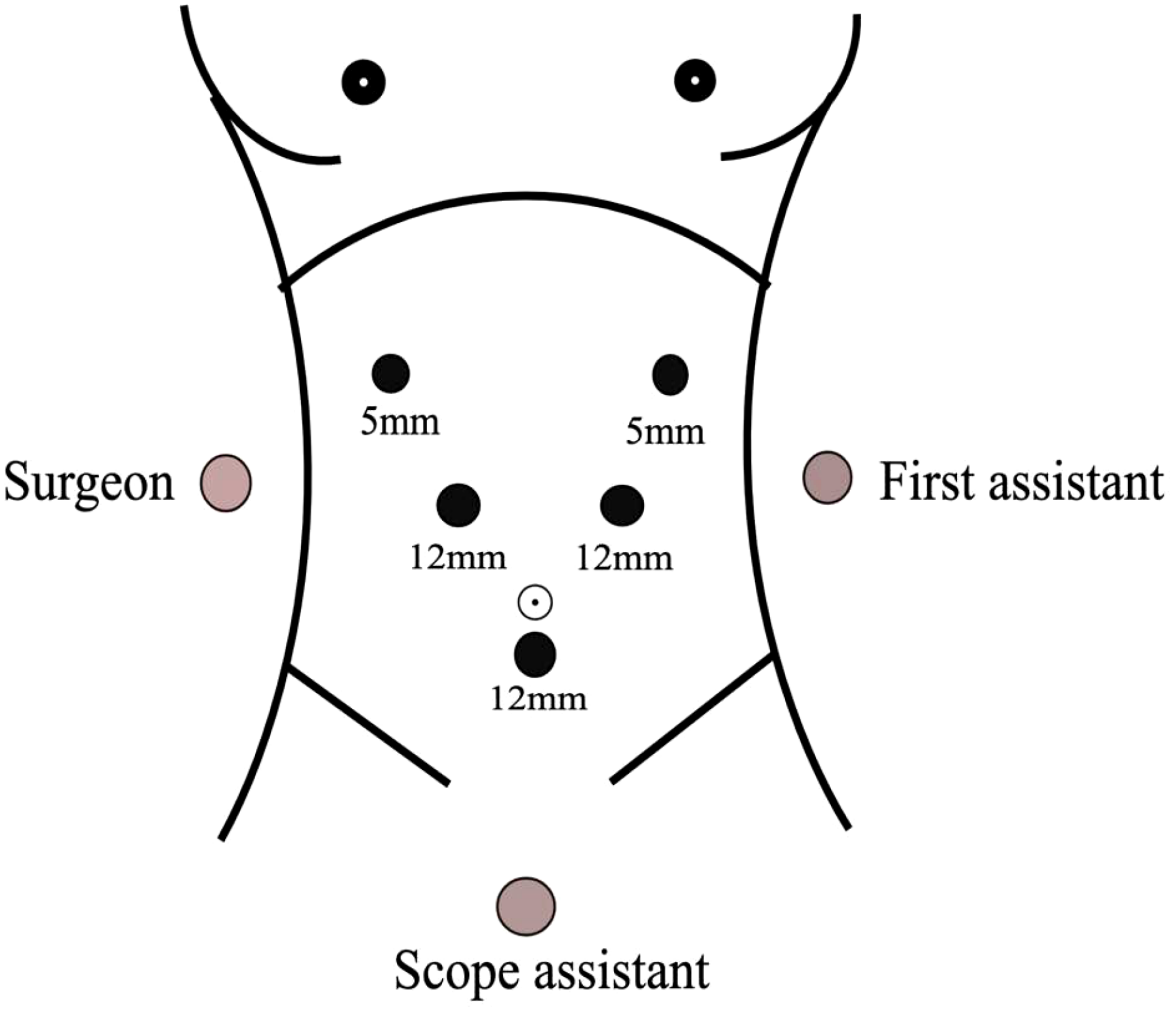
The trocar layout and the position of the patients and the operative staff.

Sizes and distribution of trocars: the observation hole was located on the umbilicus or the left edge of the umbilicus, with 5 mm and 12 mm trocar placed 1 cm below the rib edge on the right midclavicular line and 2 cm above the umbilicus on the right parasternal line, and 12 mm and 5 mm trocar placed 1cm below the rib edge on the left midclavicular line and left anterior axillary line, respectively, presenting a “V-shaped” layout. This can also be referred to that of the laparoscopic pancreaticoduodenal Trocar (**Figure 1**).

**Figure 2:**
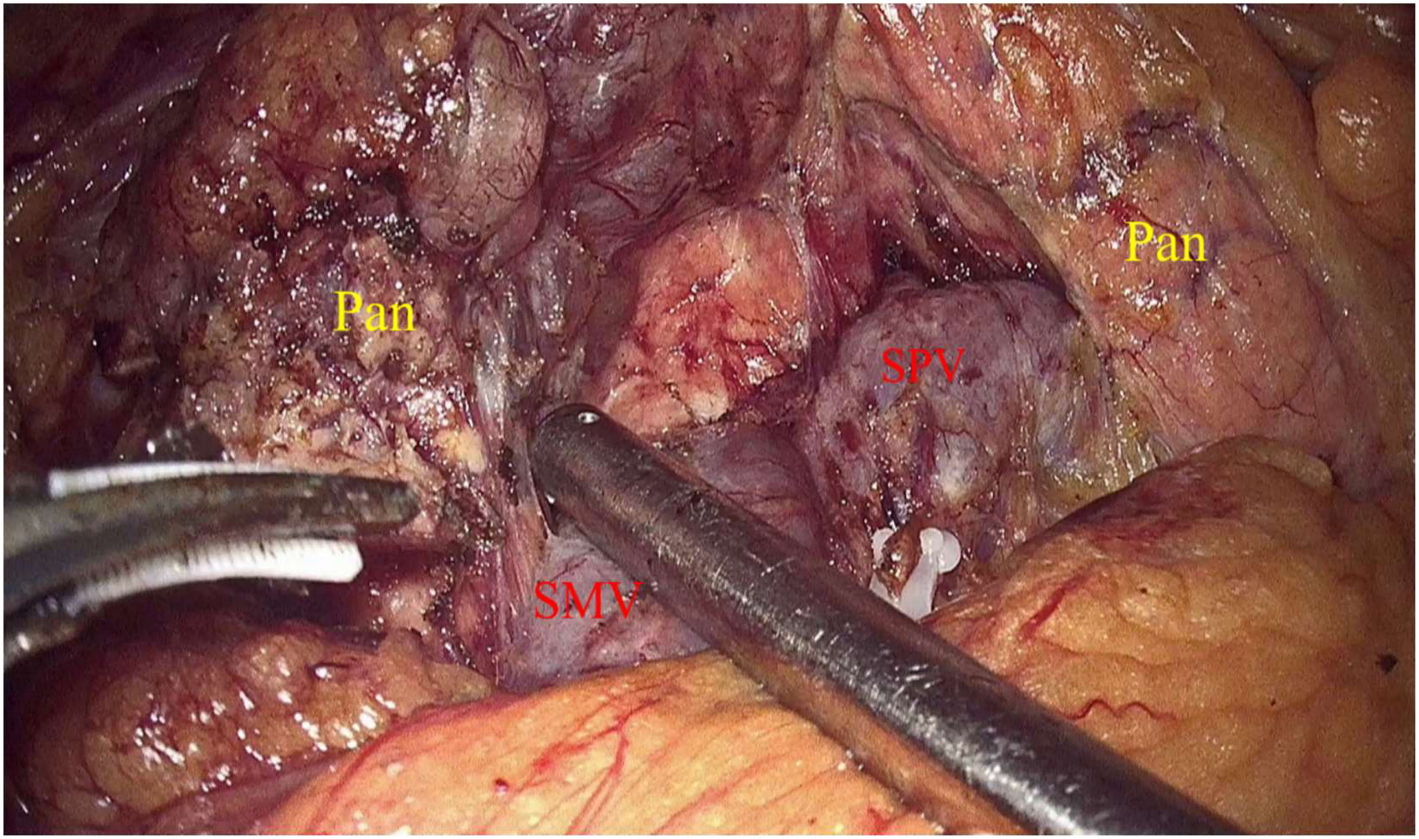
Pan, Pancreas; SMV, Inferior mesenteric vein; SPV, Splenic vein.

**Figure 3:**
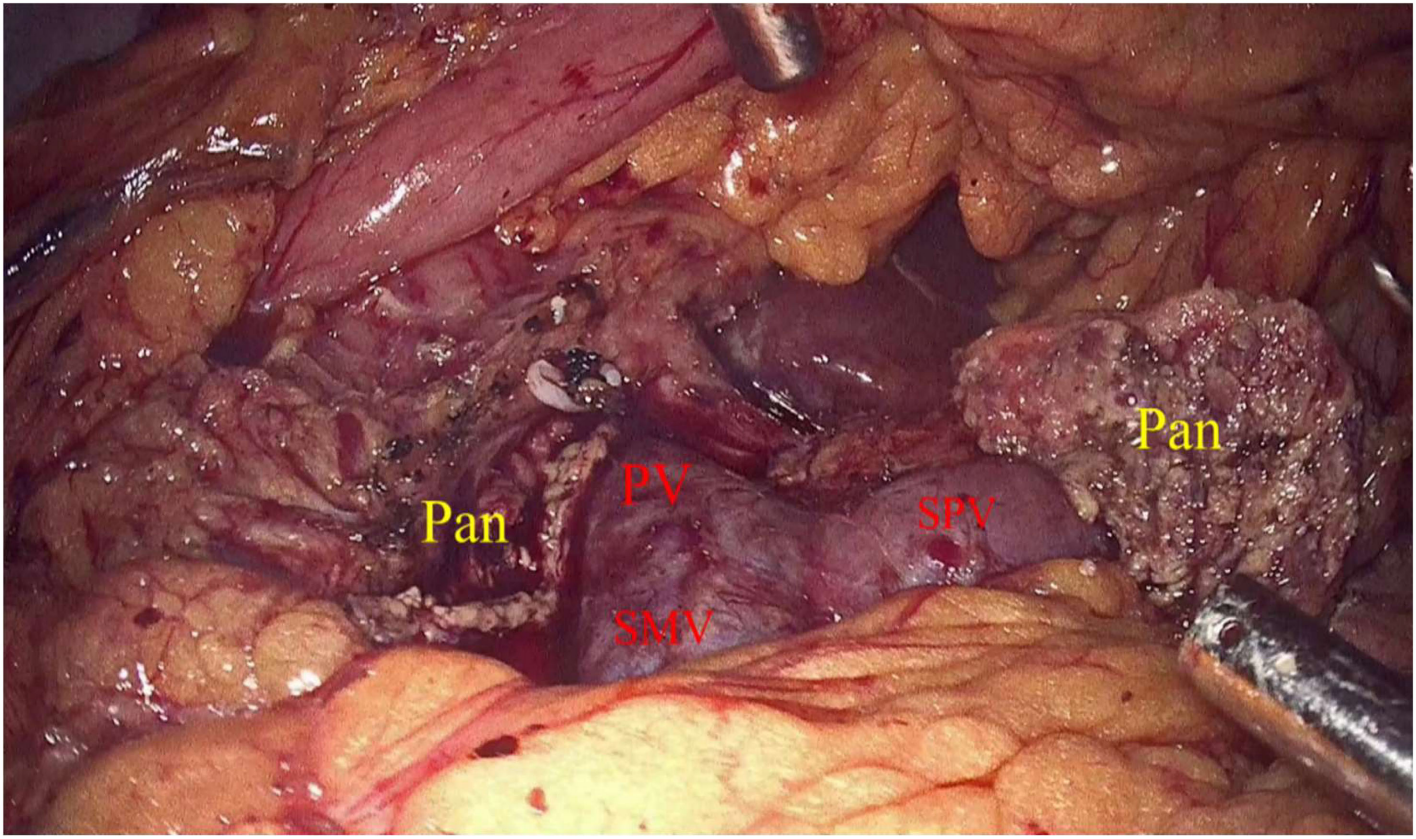
Pan, Pancreas; SMV, Inferior mesenteric vein; SPV, Splenic vein.

**Figure 4:**
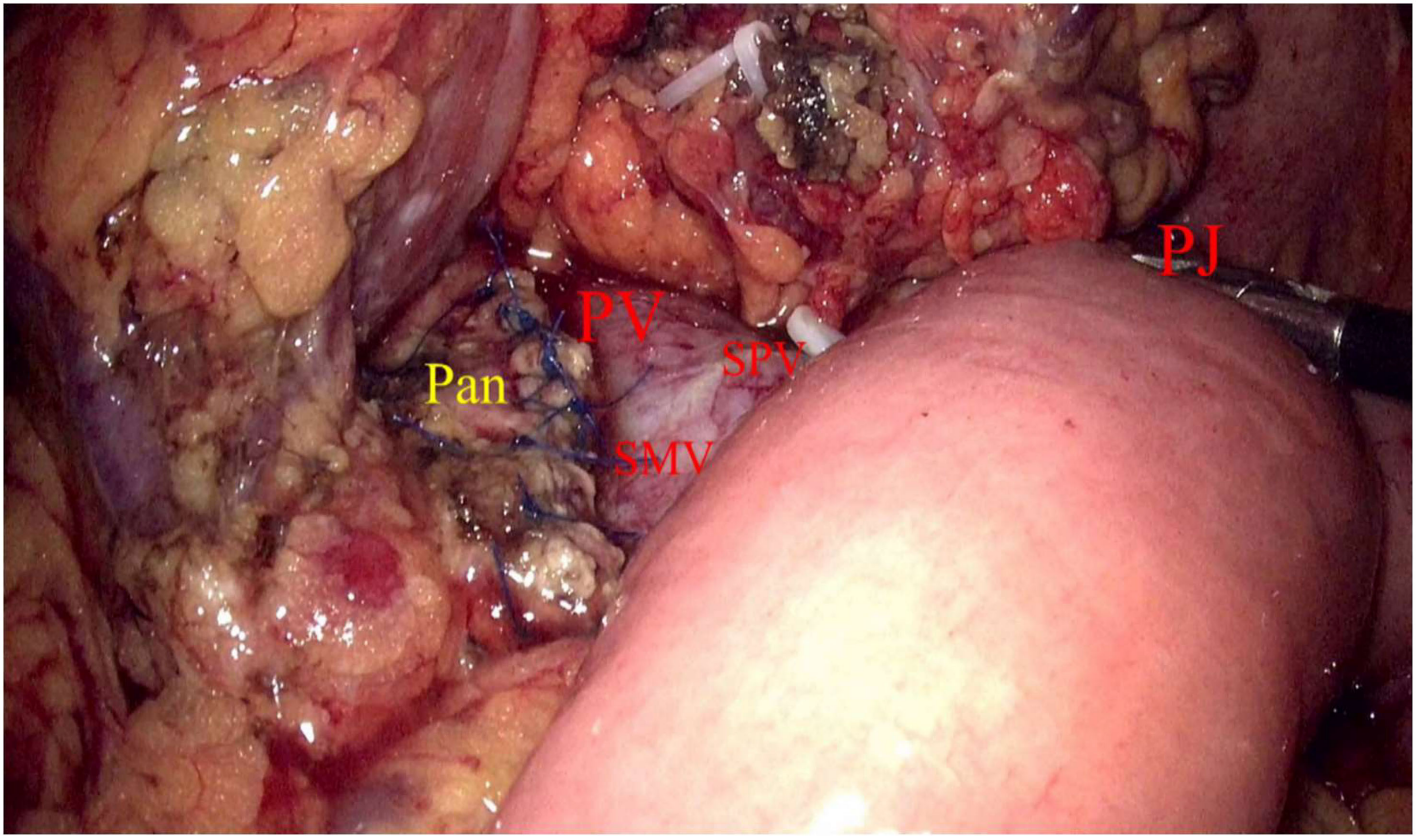
Pan, Pancreas; PV, Portal vein; SMV, Inferior mesenteric vein; SPV, Splenic vein; PJ, Pancreatic–jejunostomy.

Laparoscopic exploration and intraoperative evaluation:the abdominal cavity for metastases was routinely explored, and the gastrocolic ligament was opened to fully reveal the pancreas. In addition, the preoperative imaging results, preoperative 3D imaging, and intraoperative ultrasound would be combined to determine the location of the tumor if necessary. The proximal pancreatic tumor was cut off using a cutter after suspension of the pancreatic neck, and the middle part of the pancreas along the splenic vessels was separated to the distal end of the tumor. The pancreas was transected with an ultrasonic knife 2 cm from the distal end of the tumor (**Figure 2**). The pancreatic duct was dissected at the pancreatic duct using lumpectomy scissors, and the specimen was removed by enlarging the umbilical incision and sent for intraoperative frozen pathology to exclude malignancy. Meanwhile, 4-0 or 5-0 Prolene sutures were interrupted to close the proximal pancreatic section and the pancreatic section (**Figure 3**). The distal pancreatic duct was supported by the insertion of an appropriately sized silicone tube, and a pancreatic-jejunostomy was performed (**Figure 4**). A flushable drainage tube was placed next to each of the pancreatic-enteric anastomosis and the proximal pancreatic stump. In all cases, intraoperative frozen sections were performed to confirm negative margins, and the excised specimens were sent for pathological examination.

### Postoperative management

Antibiotics, proton pump inhibitors, growth inhibitor analogs, water-electrolyte balance, and nutrition were adopted for postoperative treatment to support the therapy. Growth inhibitor analogs were pumped intravenously for the first 5 days after surgery. Analyze abdominal drainage fluid for amylase to identify pancreatic fistulas on routine postoperative Days 1, 3, 5, and 7. Total bilirubin levels in the drainage fluid were measured for suspected biliary leakage. In the absence of clinically relevant POPF (CR-POPF) and bile leak, gradual removal of the drainage tube was usually carried out on Day 7. The nasogastric tube was removed when gastrointestinal function was restored, judged generally by anal venting, and an oral liquid diet was adopted. Nevertheless, it is necessary to be cautious about the occurrence of coeliac leakage.

### Data collection and research results

Perioperative data, including operative time, intraoperative blood loss, postoperative blood glucose, length of hospital stay, reoperations, readmissions, morbidity, and mortality (within 30 days after surgery), were collected and analyzed. POPF, delayed gastric emptying (DGE), and post-pancreatic resection hemorrhage (PPH) were assessed according to the definitions proposed by the International Study Group on Pancreatic Surgery (5–7). Grades B and C pancreatic fistulas were defined as CR-POPF. Intra-abdominal infection or abscess was diagnosed for the presence of signs of peritonitis, increased white blood cell count, and positive drainage fluid culture, which might also be detected by abdominal puncture and CT scan (8).

### Follow-up visits

All patients were followed up every 3 to 6 months in the outpatient clinic or by telephone. Long-term outcomes included pancreatic endocrine and exocrine function as well as tumor recurrence. Pancreatic endocrine insufficiency was defined as new onset diabetes mellitus (NODM) or worsening of previous diabetes mellitus, and NODM was diagnosed according to the criteria proposed by the World Health Organization (9). Generally, patients presenting with symptoms of diarrhea, steatorrhea, or weight loss requiring pancreatic enzyme replacement therapy (PERT) and/or abnormal pancreatic exocrine function tests (using fecal elastase assay) are considered to suffer from pancreatic exocrine insufficiency (PEI) (10).

### Statistical analysis

Herein, data were analyzed using SPSS25.0 for windows (SPSS, Chicago, IL). Quantitative data were expressed as mean ± standard deviation, while qualitative data were expressed as numbers and percentages.

## Results

### Perioperative results

The mean operative time was 393.2 ± 57.9 min (range: 225–497 min), the amount of intraoperative bleeding was 211.7 ± 113.9 ml (range: 50–500 ml), and the period for postoperative hospital stay was 19.4± 7.6 days (range: 8–37 days). In addition, nine cases of grade A pancreatic leak (52%), three cases of grade B pancreatic leak (17%), and no grade C pancreatic leak were observed among 17 cases. Additionally, there were no cases of abdominal infection, gastric emptying disorder, postoperative bleeding, bile leak, reoperation, or death. (**Table 2**: postoperative complications).

**Table 2:** Postoperative complications.

Variables	*n* = 17
Operative time (min)	392.2 ± 57.9
Intraoperative blood loss (ml)	211.7 ± 113.9
Morbidity (*n*/%)	12/17
Tumor size (cm)	3.4 ± 1.2
Biochemical leakage (*n*)	9
Grade B POPF (*n*)	3
DGE (*n*)	0
PPH (*n*)	0
Intra–abdominal infection/abscess (*n*)	0
Biliary leakage	0
Reoperation (*n*)	0
Mortality (*n*)	0
Postoperative hospital stays (d)	19.2 ± 7.6

### Endocrine function

Among the 17 cases, one of them was diabetic. By monitoring random blood glucose, the postoperative random blood glucose for the first 3 days was 7.4 ± 1.0 mmol/l in all cases. In comparison, the highest random blood glucose was 8.1 ± 1.0 mmol/l on the first day postoperatively, and that was 11.3 mmol/l in diabetic patients. The monitored fasting and three postprandial glucose 3 days after feeding was 6.8 ± 1.0 mmol/l. In comparison, the highest glucose on the first day after feeding was 6.8 ± 1.2 mmol/l, and the highest random glucose among them was 12.1 mmol/l, which was still a random postprandial glucose in diabetic patients. In this thesis, no insulin treatment or change in the original glucose-lowering regimen was performed in any of the above cases with elevated blood glucose, and no endocrine dysfunction was observed. No new diabetes mellitus or aggravation of diabetes mellitus was observed in the follow-up after discharge from the hospital.

### Exocrine function

Postoperative examination of formed fecal pancreatic elastase I (PEI) was 479.6 ± 114.3 µg/g feces (296.4–677.5 µg/g feces), and all patients did not show a decrease in PEI or present any exocrine dysfunction such as diarrhea. In addition, there was no re-hospitalization or tumor recurrence during the follow-up period. (**Table 3**: long-term complications).

**Table 3:** Long–term complication.

Variables	*N* = 17
Endocrine insufficiency	0
NODM	0
Worsening previous DM	0
Exocrine insufficiency	0
Recurrence	0
Follow–up periods (M)	28.5 ± 20.5

## Discussion

With the development of imaging techniques, an increasing number of benign and low-grade malignant tumors of the pancreas are being identified, and MP resection seems to be more suitable for the abovementioned lesions than PD with PD or even expanded PD, which preserves more normal pancreatic tissues. Although MP is considered to have a high incidence of POPF as well as a high bleeding rate, it is indeed an acknowledged technique for pancreatic resection, and results in long-term outcomes showing good endocrine function are especially encouraging. Since Baca and Bokan reported the first application of laparoscopic MP for the treatment of chronic pancreatitis in 2003 (11), the relevant literature has also elaborated on the use of laparoscopic, robotic MP for the treatment of benign and junctional tumours in the neck and body of the pancreas (3, 12–17). Although MP is a surgical procedure for benign or low-grade malignant tumors located in the neck or body of the pancreas, and seems to be more reasonable for benign tumors, for low-grade malignant or malignant tendencies, for example, PanIN-3, IPMN in combination with the presence of high-risk stigmata or worrisome features, and it is important to examine the pancreatic lesions preoperatively with histological examination and end-stage dissection, as well as to examine the pancreatic lesions (18). In a study of seven recurrent cases of open MP, two patients with a final pathology of MD-IPMC had positive margins and were eventually received treatment again, whereas the BD-IPMcis had no recurrence, a result that suggests that BD-IPMN appears to be more suitable for MP (19). In addition, the application of MP in early Pancreatic ductal adenocarcinoma (PDAC) is being initially explored (20).

High incidence of complications after MP has attracted the attention from many physicians. POPF remains one of the major causes of postoperative pancreatic risk events, and its risk factors are mainly affected by the texture of the pancreas and the diameter of the pancreatic duct (21). Compared with DP and PD, MP is associated with higher rates of POPF and postoperative bleeding (22, 23). In a previous meta-analysis, the MP group also had a high rate of postoperative-related clinical pancreatic fistula and a higher incidence of postoperative bleeding compared with the DP group (24). Crippa et al. (19) carried out a study of MP with the largest sample size study at that time and observed a higher rate of pancreatic fistula and complications in MP. However, the difference was not statistically significant compared to the same group of the pancreatic corporal tail resection group, and the endocrine and exocrine insufficiency rate was significantly lower in the MP group. A single-center empirical study involving 50 cases with low-grade malignancy concluded that MP was effective in preserving cephalic and distal pancreatic remnants without significantly increasing postoperative complications compared with conventional pancreatic resection (25). In the application of laparoscopic MP, Safi Dokmak et al. (26) reported the results indicating a postoperative complication rate of 74% and a CR-POPF rate of 22%. However, the main reasons for the high incidence of pancreatic fistula after MP might include the following: (1) compared to malignant tumors, benign and junctional tumors have soft pancreatic parenchyma; (2) there is often no obstruction in benign and junctional tumors, and the pancreatic duct is not dilated and has a small diameter; (c) there are two pancreatic sections in the middle pancreatic resection.

Reconstruction of the middle pancreatic resection Gastrointestinal (GI) tract mainly includes pancreatic-intestinal anastomosis, pancreatic-gastric anastomosis and “Ω” anastomosis. However, there is no definite conclusion upon the choice of pancreatic middle resection GI reconstruction. Herein, all of the 17 cases were closed by proximal suture and distal pancreatojejunostomy (PJ). According to the criteria of pancreatic fistula (5), there were nine cases of grade A fistula, three of grade B fistula and no grade C fistula, and the incidence of pancreatic fistula was 69%, while that of CR-POPF was 17%. In addition, it is similar to the reported laparoscopic MP, and the incidence of grade C fistula was lower (27). The incidence of POPF was likewise not increased compared to open MP (24, 28). All grade B fistulas were discharged from the hospital after recovery from good drainage, drain flushing, and nutritional support therapy. In terms of nutritional support therapy, Andrew A et al. suggested that patients in the comprehensive management of pancreatic cancer should not only consider anti-inflammatory diets, dietary supplements, and lifestyle modifications but also proposed a combination of traditional Chinese and Western medicine. Although it is a complex process, it helps to some extent in the treatment as well as recovery of the patient (29). In addition to PJ, the ability of PG anastomosis to reduce the incidence of POPF was also reported in other literature. In a randomized controlled study, pancreatic gastric anastomosis and pancreatico-enteric anastomosis were shown to have no difference in the incidence of pancreatic leakage as well as postoperative effects on endocrine and exocrine function, and the operative time was less (30). What is more noteworthy is that cross-disciplinary developments have also attracted the attention of pancreatic surgeons, and a technique called the “Huscher technique” has been applied to pancreatic surgery, resulting in easier pancreatic-enteric anastomosis, a relatively low incidence of POPF, and little or no impairment of exocrine function (31).

In this paper, the operative time was 393.2 ± 57.9 min (range: 225–497 min), the amount of intraoperative bleeding was 211.7 ± 113.9 ml (range: 50–500 ml), and the period for postoperative hospital stay was 19.4 ± 7.6 days (range: 8–37 days). In a review of the reported literature on laparoscopic MP (14, 23, 32–35), the operative times were 225–480 min (median operative time: 380 min), 120–285 min (median operative time: 200 min), 350 ± 63.4 min, intraoperative bleeding 50–800 ml, 477.1 ± 388.2 ml, and postoperative hospitalization time, 13.8 ± 7.3 days. In this study, the choice of pancreatic–intestinal anastomosis possible explained the relatively long operative time (15), while the relatively low amount of intraoperative bleeding (13, 14, 33) might be attributed to the relationship between the tumor and blood vessels and the existence of vascular variants. In terms of hospitalization time, the postoperative hospitalization time was 19.2 ± 7.6 days in this paper, relatively longer compared to that reported in other literature (13), which might be related to postoperative management, including the choice of time for postoperative feeding, the choice of time for drainage tube removal, and the fact that all pancreatic fistula patients were managed in hospital and discharged after complete healing. However, no case of abdominal infection, impaired gastric emptying, postoperative bleeding, bile leak, reoperation, or death was hereby reported.

MP maximized the preservation of normal physiological function of the pancreas while performing tumor resection, resulting in better preservation of endocrine function, which was significantly lower than other types of pancreatic surgery in comparison and could improve the postoperative quality of life of the patients (1, 2, 23, 33–36). In terms of endocrine function, the persistence of NODM was observed in benign or malignant tumors after PD with similar incidence. In terms of exocrine function, the presence of PEI after sexual PD in patients with benign and malignant tumors was 25.2% and 49.1%, respectively (37). Compared with DP, MP still had a lower incidence of endocrine dysfunction (25).

Herein, one of the cases involved a diabetic patient. By monitoring random blood glucose, the postoperative random blood glucose of the patients for the first 3 days was 7.4 ± 1.0 mmol/l in all cases, while, in comparison, the highest random blood glucose was 8.1 ± 1.0 mmol/l on the first day postoperatively, and the highest random blood glucose was 11.3 mmol/l for diabetic patients. The monitored fasting blood glucose 3 days after feeding and three postprandial blood glucose was 6.8 ± 1.0mmol/l. In comparison, the highest blood glucose on the first day after feeding was 6.8 ± 1.2 mmol/l, and the highest random blood glucose was 12.1 mmol/l, which was still a random postprandial blood glucose in diabetic patients. In this paper, none of the above cases with elevated blood glucose was treated with insulin or did not change the original glucose–lowering regimen, and no endocrine dysfunction was observed, which is consistent with other reports. The higher blood glucose on the first postoperative day and the first day after feeding may be attributed to surgical stress, parenteral nutrition and the diet. In terms of exocrine function, postoperative–formed fecal PEI was checked to be 479.6 ± 114.3 µg/g feces (296.4–677.5 µg/g feces) by hospitalization, and all patients did not show any decrease in PEI or signs of exocrine dysfunction such as diarrhea. In the discharge and outpatient follow–up, all patients did not have diarrhea or oral pancreatic enzyme drug–related treatment after discharge. Laparoscopic resection of the middle part of the pancreas did not cause any significant effect on the endocrine function of the pancreas.

## Conclusions

Under strict control of clinical indications, the laparoscopic MP is proven to be safe and feasible in the treatment of benign or low–grade malignant tumors in the body of the pancreatic neck, causing no increased risk of intraoperative and postoperative complications and endocrine dysfunction of the pancreas. However, this thesis is still restricted by a limited sample size as well as a short period of case follow–up. Further breakthroughs could be made in these areas. In addition, with the emergence of artificial intelligence (38), more precise resection with preservation of parenchymal organs is expected to receive increasing attention. There will be more multi–center and large sample studies.

## Data availability statement

The datasets presented in this study can be found in online repositories. The names of the repository/repositories and accession number(s) can be found in the article/supplementary material.

## Ethics statement

The studies involving humans were approved by Hunan Provincial People’s Hospital/First Affiliated Hospital of Hunan Normal University. The studies were conducted in accordance with the local legislation and institutional requirements. The participants provided their written informed consent to participate in this study. Written informed consent was obtained from the individual(s) for the publication of any potentially identifiable images or data included in this article.

## Author contributions

YaL and WZ are mainly responsible for data analysis and paper writing and revision. YaL and MD are mainly responsible for paper revision, translation and embellishment. WZ and JZ are mainly responsible for data collection and processing analysis. YW is mainly responsible for data collection. XH is mainly responsible for paper ethics. YiL was mainly responsible for the design of the paper, data analysis, and paper revision. WC was mainly responsible for providing design input, data processing analysis, and paper revision of the paper. All authors contributed to the article and approved the submitted version.
